# Geographic Variation in Botulinum Toxin Type A Longevity: A Pilot Comparative Study Between Saint Petersburg and Ashgabat

**DOI:** 10.7759/cureus.110279

**Published:** 2026-06-05

**Authors:** Zuleyha Allambergenova

**Affiliations:** 1 Dermatology and Aesthetic Medicine, Kosmet_A Clinic, Saint Petersburg, RUS; 2 Dermatology and Aesthetic Medicine, Golli Beautique, Ashgabat, TKM

**Keywords:** aesthetic medicine clinic, a pilot study, botulinum toxin type a, dermal photoaging, ultraviolet exposure, ultraviolet radiation exposure

## Abstract

The clinical duration of botulinum toxin type A (BoNT-A) is commonly associated with anatomical factors, yet environmental influences remain an area of clinical interest. This observation, based on clinical practice in Saint Petersburg and Ashgabat, explores potential variations in neuromodulator requirements across different climatic zones. It has been hypothesized that chronic solar exposure may be associated with differences in facial muscle engagement and aesthetic treatment outcomes; however, this relationship was not directly evaluated in the present study. The purpose of this pilot investigation was to explore whether differences in BoNT-A dosage requirements and treatment longevity could be observed between two geographic cohorts residing in regions with different climatic and ultraviolet (UV) environments. Some patients underwent platelet-rich plasma (PRP) therapy or biorevitalization as part of routine clinical practice prior to BoNT-A administration. However, the present study was not designed to evaluate the effectiveness of these interventions. These observations are intended to highlight the need for further investigation of potential climate-related factors influencing aesthetic treatment outcomes.

## Introduction

Aesthetic medicine often underestimates the geographic context of the patient [1]. While facial kinetics are well-understood, the influence of regional climate remains insufficiently studied [2]. Clinical observations in different geographic regions have raised the question of whether environmental conditions may influence aesthetic treatment outcomes. However, the relationship between environmental factors and botulinum toxin type A (BoNT-A) outcomes remains insufficiently studied [3]. Furthermore, arid environments may contribute to increased transepidermal water loss and skin changes associated with photoaging [4,5]. Whether such factors influence BoNT-A outcomes remains uncertain and requires further investigation. Given the limited available data, the potential influence of geographic and environmental factors on BoNT-A outcomes remains an area for further study. The primary objective of this pilot study was to explore whether differences in botulinum toxin type A dosage requirements and treatment longevity could be observed between two geographic cohorts residing in regions with different climatic and UV environments. This study was intended as an exploratory, hypothesis-generating investigation rather than a causal analysis of UV exposure.

## Materials and methods

Study design and period

This retrospective comparative pilot study was conducted over an 18-month period (January 2024 - June 2025). The study evaluated clinical outcomes of botulinum toxin type A (BoNT-A) injections in two distinct geographic cohorts: Saint Petersburg, Russia (Low UV Index: 2-3) and Ashgabat, Turkmenistan (High UV Index: 7-9). Regional UV index values were derived from climatological averages for each study location and were used as geographic descriptors. Individual UV exposure was not measured. Information regarding individual sun exposure, sunscreen use, smoking status, occupational exposure, and skin phototype was not consistently available in the medical records and, therefore, could not be included in the analysis.

Inclusion and exclusion criteria

The inclusion criteria were (1) patients aged 20-50 years; (2) primary diagnosis of dynamic glabellar or forehead rhytides; (3) residence in the respective geographic zone for at least two years. The exclusion criteria were (1) hypersensitivity to neurotoxins; (2) systemic neuromuscular disorders; (3) facial surgery or thread lifting within the last 12 months; (4) pregnancy or lactation.

Treatment procedures

All botulinum toxin type A treatments were performed as part of routine clinical practice by experienced aesthetic physicians at the participating clinics. Injection sites and dosing were determined according to standard clinical assessment of dynamic glabellar and forehead rhytides. The same botulinum toxin type A formulation (onabotulinumtoxinA, Botox) was used in both participating clinics throughout the study period. Because of the retrospective nature of the study, treatment protocols were not prospectively standardized between centers.

Outcome assessment

Treatment longevity was defined as the interval between the initial treatment and the first documented clinical observation of return of dynamic muscle activity requiring repeat treatment. Dosage requirements were extracted from medical records and recorded in units administered during the treatment session.

Statistical analysis

Statistical analysis was performed using IBM SPSS Statistics version 27.0 (IBM Corp., Armonk, NY, USA). Data are presented as mean ± standard deviation (SD) and frequencies (N, %). Differences between groups were analyzed using the Independent Samples t-test. A p-value < 0.05 was considered statistically significant. Because this was an exploratory pilot study, no formal sample size calculation or statistical power analysis was performed.

Ethical details

The study was conducted in accordance with the Declaration of Helsinki. This retrospective study involved analysis of de-identified clinical data and did not include any prospective intervention or direct patient contact. Formal ethical approval was not required according to institutional policies governing retrospective analysis of anonymized data, and the study complied with applicable local regulations. Written informed consent for publication of clinical photographs was obtained from all patients.

## Results

Patient demographics

A total of 24 patients (N=24) were included in this pilot study, divided into two equal geographic cohorts: the low-UV cohort from Saint Petersburg (n=12; 50%) and the high-UV cohort from Ashgabat (n=12; 50%). The mean age across both groups was approximately 37 years, with no statistically significant difference between the cohorts (p > 0.05). Baseline demographic and clinical characteristics are summarized in Table 1.

**Table 1:** Baseline demographic and clinical characteristics of the study population (N=24) Data are presented as Mean ± SD and n (%). Statistical comparisons between groups were performed using the Independent Samples t-test. Test statistic values (t) and corresponding p-values are presented. * p < 0.05 was considered statistically significant. BoNT-A: botulinum toxin type A

Parameter	Saint Petersburg (n=12; 50%)	Ashgabat (n=12; 50%)	Test statistic (t)	p-value
Mean age (years ± SD)	36.3 ± 5.7	37.5 ± 6.2	0.49	0.63
Female patients, n (%)	9 (75%)	8 (66.7%)	0.21	0.65
Male patients, n (%)	3 (25%)	4 (33.3%)	0.21	0.65
Mean BoNT-A dose (Units ± SD)	19.0 ± 1.9	23.7 ± 2.0	5.9	<0.001*
Mean duration of effect (days ± SD)	107.8 ± 8.7	79.3 ± 9.4	7.7	<0.001*
Patients receiving skin preparation, n (%)	5 (41.7%)	5 (41.7%)	0.00	1.00
Mean UV Index	2–3	7–9	N/A	N/A

Comparison of BoNT-A dosage requirements

Analysis of treatment protocols revealed that patients residing in the high-UV region (Ashgabat) required a higher mean dose of botulinum toxin type A to achieve the desired aesthetic effect compared to those in the low-UV region (Saint Petersburg). Specifically, the mean dose in the Ashgabat group was 23.7 ± 2.0 Units, while the Saint Petersburg group required a mean dose of 19.0 ± 1.9 Units. This difference was statistically significant (t = 5.9, p < 0.001), suggesting a difference between the two geographic cohorts evaluated in this pilot study.

Duration of clinical effect

**Figure 1:**
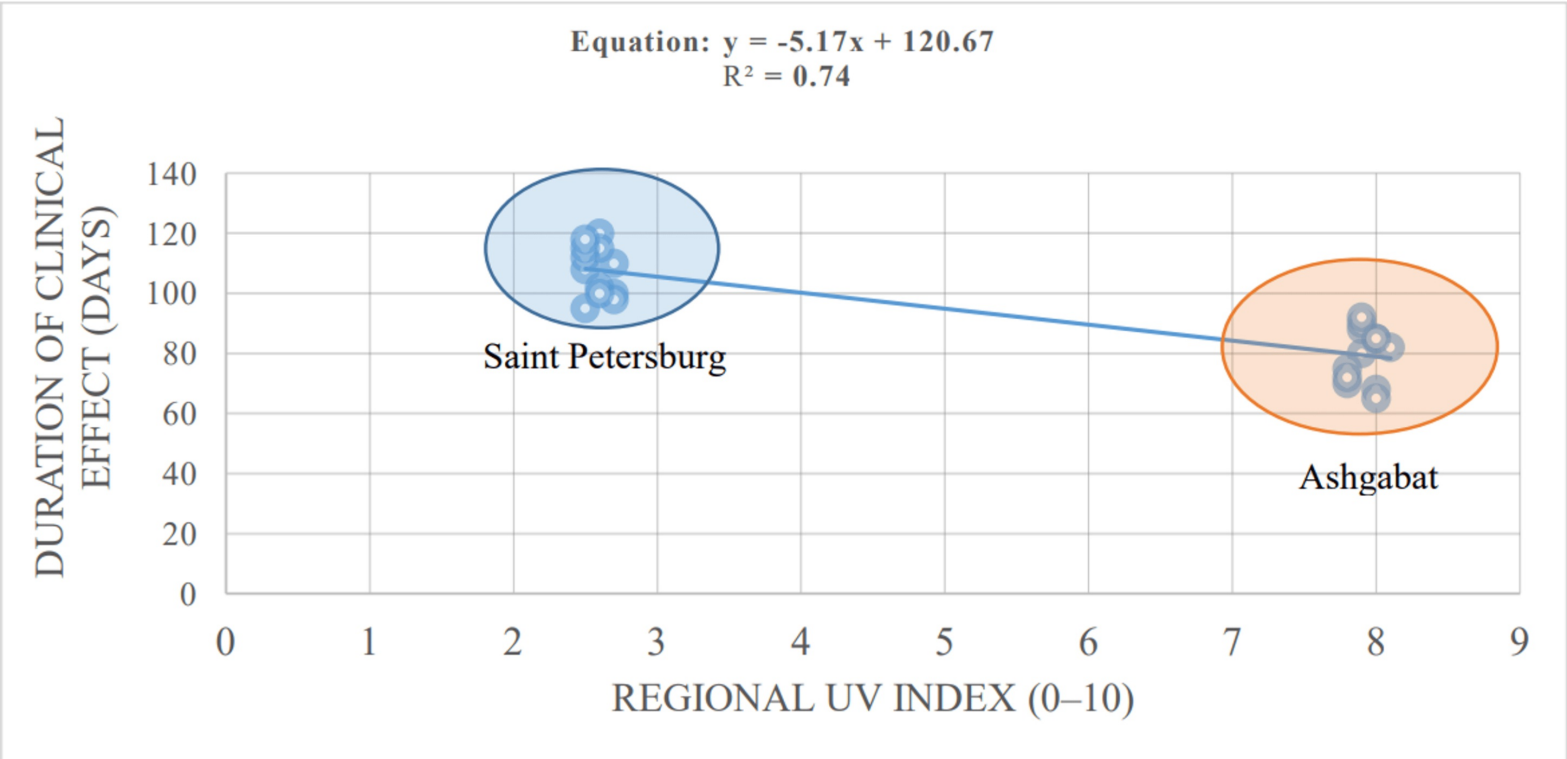
Scatter plot demonstrating the relationship between regional UV index values and observed BoNT-A effect duration in the two geographic cohorts Regional UV index values were used as geographic descriptors and do not represent individual patient UV exposure. BoNT-A: botulinum toxin type A

The longevity of the BoNT-A effect differed between the two geographic cohorts. The duration of effect in the high-UV cohort was markedly shorter, averaging 79.3 ± 9.4 days. In contrast, the low-UV cohort experienced a mean duration of 107.8 ± 8.7 days. Statistical analysis demonstrated a statistically significant reduction in longevity (t = 7.7, p < 0.001). An exploratory regression analysis demonstrated a negative relationship between regional UV index values and observed BoNT-A effect duration within the two study cohorts (R² = 0.74). Because only two geographic cohorts were included, this analysis should be interpreted as descriptive and hypothesis-generating. These results are further detailed in Table 2, Table 3, and Figure 1.

**Table 2:** Comparative analysis of BoNT-A outcomes (N=24) Data are represented as Mean ± SD. Statistical comparisons were performed using the Independent Samples t-test. * p < 0.05 was considered statistically significant. BoNT-A: botulinum toxin type A

Parameter	Saint Petersburg (n=12, 50%)	Ashgabat (n=12, 50%)	Test statistic (t)	p-value
Mean dose (Units ± SD)	19.0 ± 1.9	23.7 ± 2.0	5.9	<0.001*
Duration of effect (Days ± SD)	107.8 ± 8.7	79.3 ± 9.4	7.7	<0.001*

**Table 3:** Influence of pre-treatment skin preparation on the duration of BoNT-A clinical effect Data are represented as Mean ± SD. Statistical comparisons were performed using the Independent Samples t-test. * p < 0.05 was considered statistically significant. Pre-treatment skin preparation included platelet-rich plasma (PRP) and/or biorevitalization administered 14 days prior to BoNT-A injection. BoNT-A: botulinum toxin type A

Parameter	Without skin preparation (n=14; 58.3%)	With skin preparation (n=10; 41.7%)	Test statistic (t)	p-value
Mean duration (Days ± SD)	92.0 ± 12.0	105.0 ± 10.0	1.94	0.07*

Clinical cases

Case 1 (Low UV)

**Figure 2:**
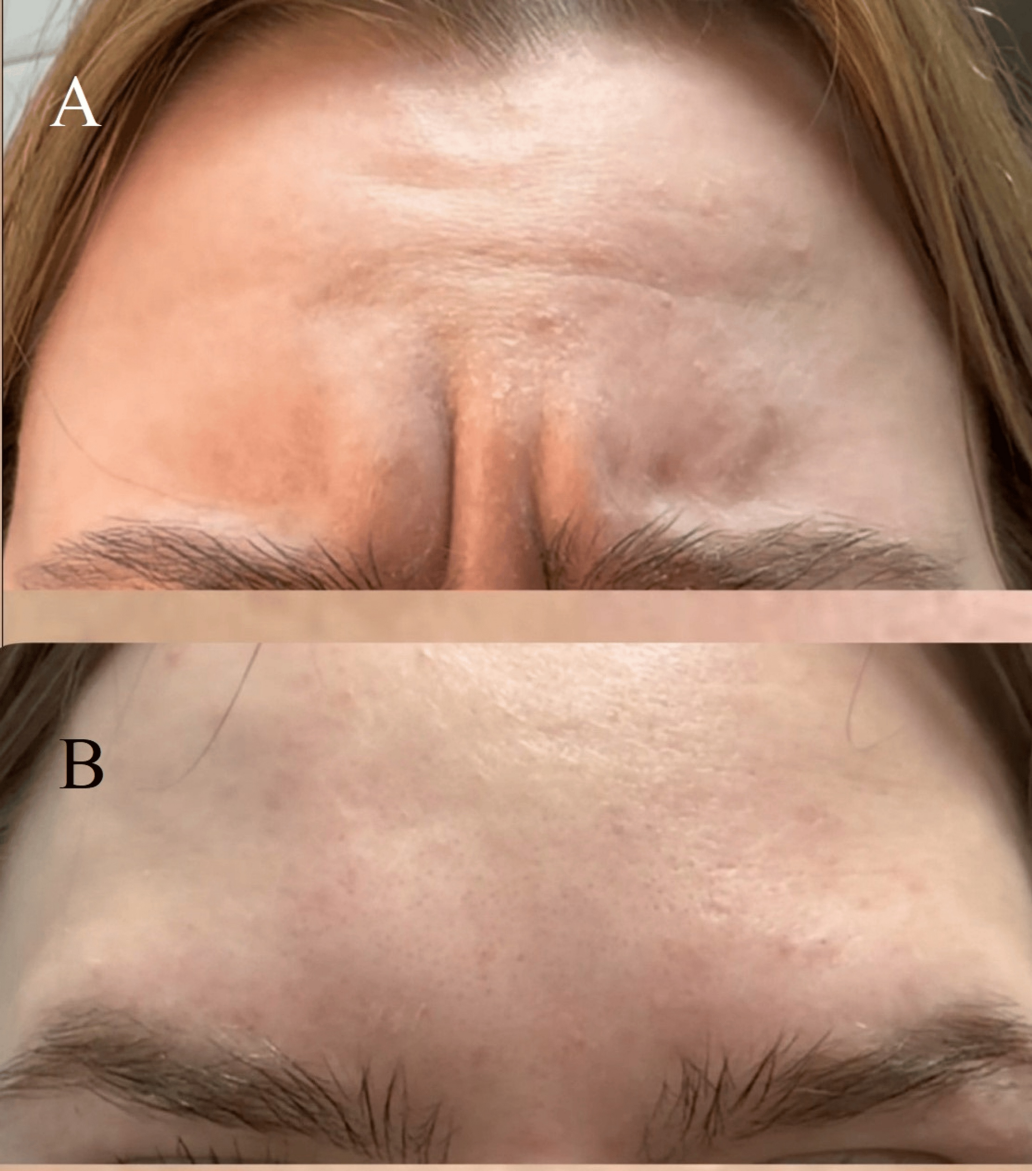
Clinical outcomes in a patient from a low-UV region (A) Baseline appearance prior to botulinum toxin type A injection demonstrating dynamic glabellar rhytides. (B) Follow-up image obtained approximately 5.5 months after treatment, demonstrating preserved aesthetic effect and reduction of dynamic muscle activity.

A 24-year-old female patient presented with dynamic glabellar lines and a predisposition to edema (Figure 2). The patient underwent full-face treatment (38 U), exceeding the study mean for localized zones. The patient demonstrated a stable clinical response with preserved facial dynamics and no adverse effects. The duration of effect was approximately 5.5 months.

Case 2 (High UV Region - Ashgabat)

**Figure 3:**
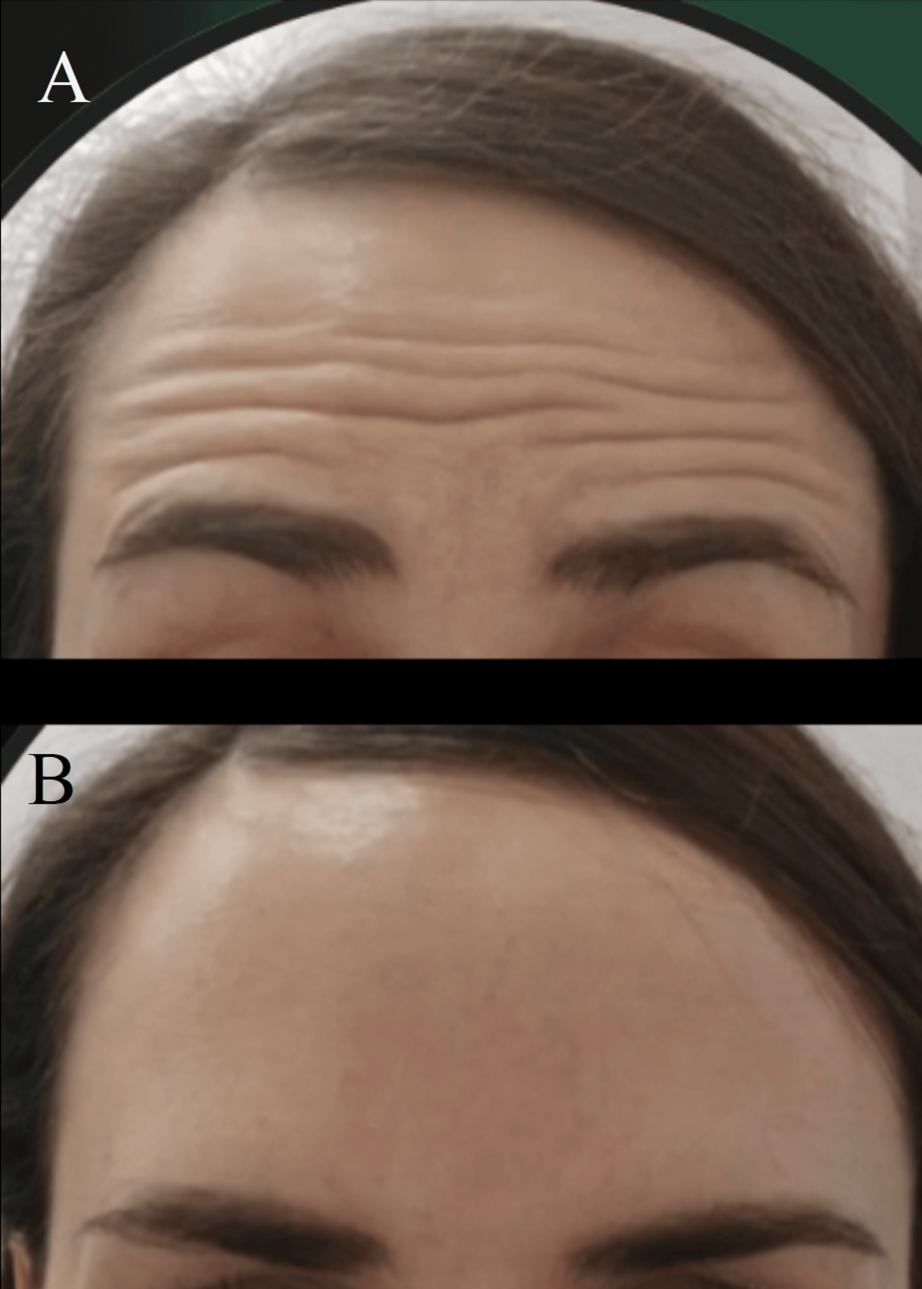
Clinical outcomes in a patient from a high-UV region (A) Baseline appearance demonstrating periorbital rhytides and signs of photoaging prior to treatment. (B) Follow-up image after combined platelet-rich plasma (PRP), biorevitalization, and botulinum toxin type A therapy demonstrating improvement in skin texture and maintained clinical response.

A 41-year-old female patient presented with periorbital rhytides and signs of photoaging (Figure 3). A combined protocol (PRP, biorevitalization, and BoNT-A 44 U) was used. The present study was not designed to evaluate the effectiveness of these interventions. The duration of effect was approximately five months despite high environmental temperatures.

## Discussion

The results of this pilot study demonstrated differences in BoNT-A outcomes between two geographic cohorts from Saint Petersburg and Ashgabat. Patients residing in Ashgabat demonstrated higher dosage requirements and a shorter duration of effect compared to the cohort in Saint Petersburg. Ashgabat is characterized by a higher regional UV Index (7-9) and greater solar exposure than Saint Petersburg; however, individual UV exposure was not assessed in this study. One possible explanation for the observed geographic differences may involve behavioral responses to environmental conditions; however, facial muscle activity was not directly measured in this study, and no mechanistic conclusions can be drawn [3]. Furthermore, chronic UV exposure is known to contribute to photoaging, dermal matrix degradation, and increased oxidative stress [4,5]. Whether these factors influence BoNT-A outcomes remains uncertain and was not evaluated in the present study. Our observation that patients in Ashgabat required an average of 23.7 ± 2.0 Units compared to 19.0 ± 1.9 Units in Saint Petersburg (p < 0.001) suggests that geographic differences in treatment outcomes may warrant further investigation. Although the present findings remain observational, they suggest that geographic and environmental differences may warrant further investigation in future studies of aesthetic treatment outcomes.

Some patients underwent PRP or biorevitalization as part of routine clinical practice. The present study was not designed to evaluate the effectiveness of these interventions, and no conclusions regarding their clinical benefit can be drawn.

Limitations

The primary limitation of this study is the small sample size (N=24), which may limit the statistical power and generalizability of the findings. As a retrospective pilot study, it was not possible to control for all potential confounding variables, including the use of sunglasses, sun protection factor (SPF) products, occupational exposure, lifestyle factors, and individual metabolic differences. Regional UV exposure was estimated using climatological averages rather than individual patient exposure measurements, and no formal sample size calculation was performed. Additionally, the study focused on a specific demographic (patients aged 20-50 years), and the findings may not be applicable to other age groups. Future prospective multicenter studies with larger cohorts and standardized assessment of individual UV exposure would be valuable to further evaluate these preliminary observations and their potential clinical implications.

## Conclusions

This pilot study identified differences in BoNT-A outcomes between two geographic cohorts. Because individual UV exposure was not measured and potential confounding factors were not fully controlled, the findings should be considered exploratory and hypothesis-generating. Larger prospective studies are needed to clarify the factors contributing to these regional differences.
